# Early identification of PRISm from chest CT: a multimodal for three-class stratification of normal, PRISm, and COPD

**DOI:** 10.3389/fradi.2026.1829853

**Published:** 2026-07-01

**Authors:** Yan Mou, Xueqing Bian, Na Tian, Zheng Xia, Jinbo Shi, Ming Li, Zhimin Shao, Lin Qi, Haiyan Ge, Miao Huang

**Affiliations:** 1 Department of Pulmonary and Critical Care Medicine, Huadong Hospital, Fudan University, Shanghai, China; 2 School of Computer and Information Engineering, Shanghai Polytechnic University, Shanghai, China; 3 Department of Pulmonary and Critical Care Medicine, Kaiyuan People's Hospital, Kaiyuan, Yunnan, China; 4 Department of Pulmonary and Critical Care Medicine, Jinggu County People's Hospital, Pu'er, Yunnan, China; 5 Department of Radiology, Jinggu County People's Hospital, Pu'er, Yunnan, China; 6 Department of Radiology, Huadong Hospital, Fudan University, Shanghai, China; 7 Huadong Hospital, Fudan University, Shanghai, China; 8 School of Intelligent Medical and Health Engineering, Shanghai Polytechnic University, Shanghai, China

**Keywords:** attention-driven, chronic obstructive pulmonary disease, multi-instance learning (MIL), multimodal, preserved ratio impaired spirometry

## Abstract

**Objective:**

Preserved ratio impaired spirometry (PRISm), a precursor to chronic obstructive pulmonary disease (COPD), is difficult to diagnose, primarily due to its subtle and inconspicuous changes in lung structure. This study aims to develop an attention-driven multi-instance learning framework with ResNet34 (MIL-R34) that integrates CT imaging with clinical data to automatically classify normal, PRISm, and COPD, thereby addressing current limitations in early PRISm detection.

**Materials and Methods:**

A total of 1,063 participants from two centers who underwent thin-slice chest CT (<1 mm) and pulmonary function tests within 2 weeks were retrospectively analyzed. After screening, 966 participants were included (Center A: 776; Center B: 190 for external testing). Participants were divided into normal, PRISm, and COPD groups. For each subject, 20 CT slices were selected and processed using a denoising strategy. Four deep learning models (ResNet34, CNN, Swin Transformer, EfficientNet) were compared. Clinical data were encoded and combined with imaging features to construct a multimodal model. Performance was evaluated using AUC, accuracy, sensitivity, and specificity.

**Results:**

Among CT-only models, ResNet34 achieved the best performance (AUC 0.873, accuracy 0.699). The clinical-only model showed limited performance (AUC 0.656, accuracy 0.423), whereas the multimodal model further improved performance (AUC 0.879, accuracy 0.724), outperforming both unimodal approaches. External validation yielded an AUC of 0.808 and an accuracy of 0.642, demonstrating reasonable generalizability despite performance decline across centers.

**Conclusion:**

This study demonstrates that chest CT imaging, when combined with clinical demographic data, can reliably identify PRISm as a transitional state between normal lung function and COPD. Furthermore, the proposed multimodal provides interpretable imaging biomarkers that enhance COPD risk stratification and address current diagnostic limitations in the early detection of PRISm.

## 1 Introduction

Chronic obstructive pulmonary disease (COPD), characterized by persistent airflow limitation, remains a leading global cause of morbidity and mortality. Nowadays, the early identification of impaired lung function has become increasingly concerned. Preserved ratio impaired spirometry (PRISm), is defined as the forced expiratory volume in 1 s (FEV_1_) < 80% of predicted value, even though FEV_1_/forced vital capacity (FVC) ratio (≥0.70) is preserved. PRISm is a precursor state to COPD. Epidemiological studies report a PRISm prevalence of 5%–20% in general populations (1). PRISm has been identified as a subtype that is more susceptible to developing COPD, with 32.6% of individuals progressed to COPD within 4–5 years (2). Moreover, research from cohort studies has shown that individuals with PRISm face a higher all-cause mortality rate than those with normal lung function. They also encounter a rise in cardiovascular incidents and a markedly increased likelihood of developing systemic comorbidities (3). Therefore, identifying PRISm is crucial for early intervention and prevention strategies that can mitigate the individual and societal impact of the disease.

Pulmonary function tests (PFTs) are the gold standard for COPD and PRISm. However, their ability to detect early-stage disease is limited. Furthermore, many individuals lack awareness of PFTs and do not proactively seek testing, while others cannot tolerate or complete the required breathing maneuvers, further restricting their feasibility for early risk stratification (4). Chest CT is widely utilized not only in routine clinical practice but also in population-based health screenings, offers a noninvasive alternative for capturing these subtle anatomical changes (5, 6). However, radiologists may not always capture the subtle changes that characterize PRISm. Furthermore, these methods can be subjective and vary between different radiologists, leading to potential misclassification. Artificial intelligence technology has made breakthrough progress in the field of medical image analysis, providing new ideas to solve the above problems. Deep learning has gradually replaced traditional manual feature extraction as the mainstream technical route for chest CT imaging classification. The multi-instance learning (MIL) method performs particularly well in the task of chest CT sequence classification due to its special problem modeling approach.

However, existing studies still face three key challenges: Firstly, most prior studies have focused on binary classification tasks (e.g., normal vs. COPD, normal vs. PRISm, or COPD vs. PRISm). Deep learning models capable of simultaneously differentiating among normal, PRISm, and COPD remain lacking, highlighting a critical gap in early COPD risk prediction (7–9). Second, noise artifacts in thin-slice CT scans impair early lesion detection, with current denoising methods being inadequate for preserving disease-specific features (10).

To overcome the underdiagnosis of PRISm, this study developed an attention-driven MIL-R34 network for three-class classification. The proposed multimodal framework integrates CT image and clinical data to enhance early detection.

## 2 Materials and methods

### 2.1 Patients

The present work gained approval from ethics committees of Huadong Hospital affiliated to Fudan University (2025K133). No informed consent was needed because this was a retrospective study (ClinicalTrials.gov registration number: ChiCTR2000030911). From January 2023 to June 2024, a total of 850 participants from Huadong Hospital affiliated to Fudan University who underwent thin-slice chest CT (<1 mm) and PFTs were included. Meanwhile, a total of 213 participants from Jinggu County People's Hospital were included. They were categorized into three diagnostic groups: normal, PRISm or COPD. Medical records of all participants were reviewed, including age, sex, height, weight, body mass index (BMI) and PFTs. The collected pulmonary function measures comprised: FEV_1_ % predicted, FEV_1_/FVC, vital capacity (VC) % predicted, forced expiratory flow at 50% of FVC (FEF₅₀ % predicted), forced expiratory flow at 75% of FVC (FEF75 % predicted), forced expiratory flow between 25% and 75% of FVC (FEF₂₅₋₇₅ % predicted), total lung capacity (TLC) % predicted, residual volume (RV) % predicted, RV/TLC, diffusing capacity of the lung for carbon monoxide (DLCO) % predicted, and DLCO adjusted for alveolar volume (DLCO/VA). Chest computed tomography (CT) images were also reviewed as part of the assessment.

### 2.2 Inclusion criteria and exclusion criteria

Participants were enrolled if they met the following criteria: (1) availability of both chest CT and PFTs performed at the same institution; (2) completion of chest CT and PFTs with an interval of less than 2 week; (3) availability of thin-slice (<1 mm) chest CT images. Patients were excluded for any of the following: (1) comorbid other thoracic disease (pulmonary atelectasis, pneumonia, lung nodules >6 mm, pleural effusion, or asthma); (2) lung post-surgery; (3) presence of spinal implants or significant CT artifacts affecting lung parenchyma visualization; (4) lack of complete clinical data.

A total of 850 participants were initially screened. After applying the inclusion and exclusion criteria, 776 participants were finally included in the study. For model construction and validation, participants were randomly assigned to the training (60%), validation (20%), and test (20%) sets using a stratified sampling strategy to ensure balanced group distribution.

In addition, an independent external cohort was established. A total of 213 participants who underwent both chest CT and pulmonary function tests between January 2025 and June 2026 were initially enrolled. After excluding cases with incomplete clinical data (*n* = 11), failed pulmonary function tests (*n* = 8), and poor-quality CT images (*n* = 4), 190 participants were ultimately included as the external test set.

**Figure 1:**
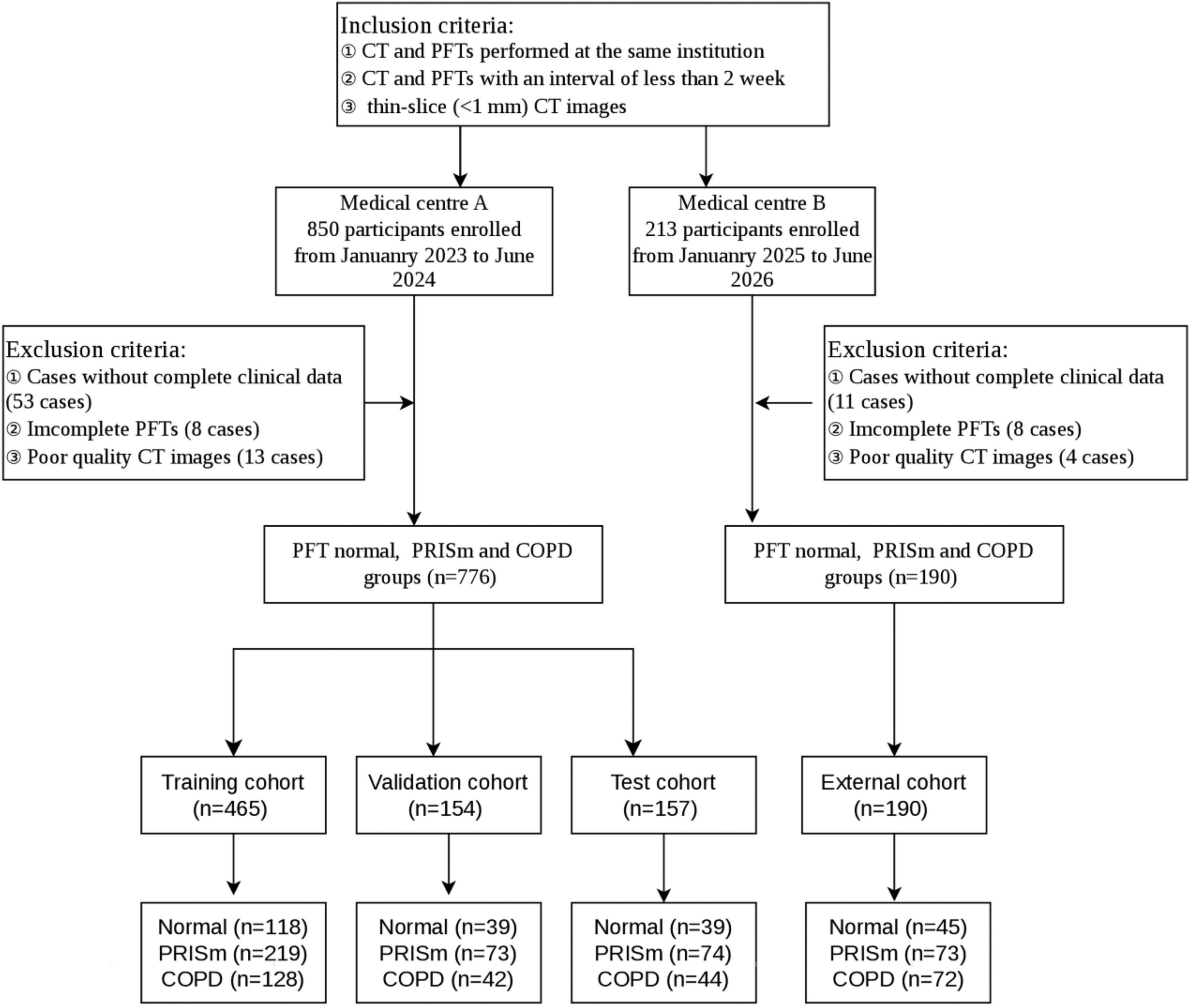
Participants recruitment process, inclusion and exclusion decision tree. PRISm, preserved ratio impaired spirometry; COPD, chronic obstructive pulmonary disease.

The overall patient recruitment process is illustrated in Figure 1.

### 2.3 Acquisition of chest CT images and PFTs

Chest CT scans were performed using GE LightSpeed VCT 64-slice and Siemens Somatom Definition Flash. The scanning parameters included a tube current ranging from 80 to 140 mA, a tube voltage between 80 and 120 kV, and a slice thickness of 1 mm. Scans were acquired in the full inspiration phase.

PFTs were obtained from a Jaeger Toennies spirometer (Höchberg, Germany) according to the American Thoracic Society (ATS) guidelines (11). The parameters including FEV1 % predicted and FEV1/FVC were recorded. PFTs were performed by professional technicians and the results were interpreted by two physicians. FEV1/FVC < 0.7 after bronchodilator use were diagnosed as COPD. Patients with FEV1/FVC ≥ 0.70 and FEV_1_ ＜ 80% were deemed as PRISm.

### 2.4 Chest CT dataset preprocessing

The chest CT dataset included three categories: normal, PRISm, and COPD cases. The preprocessing pipeline consisted of multiple sequential steps designed to improve image quality and enhance the robustness of feature extraction. First, 20 representative axial CT slices were selected for each participant to ensure adequate coverage of the entire lung region. Subsequently, CT values were standardized using optimized window width (1,500) and window level (−450) settings to improve contrast between pulmonary parenchyma and surrounding anatomical structures.

**Figure 2:**
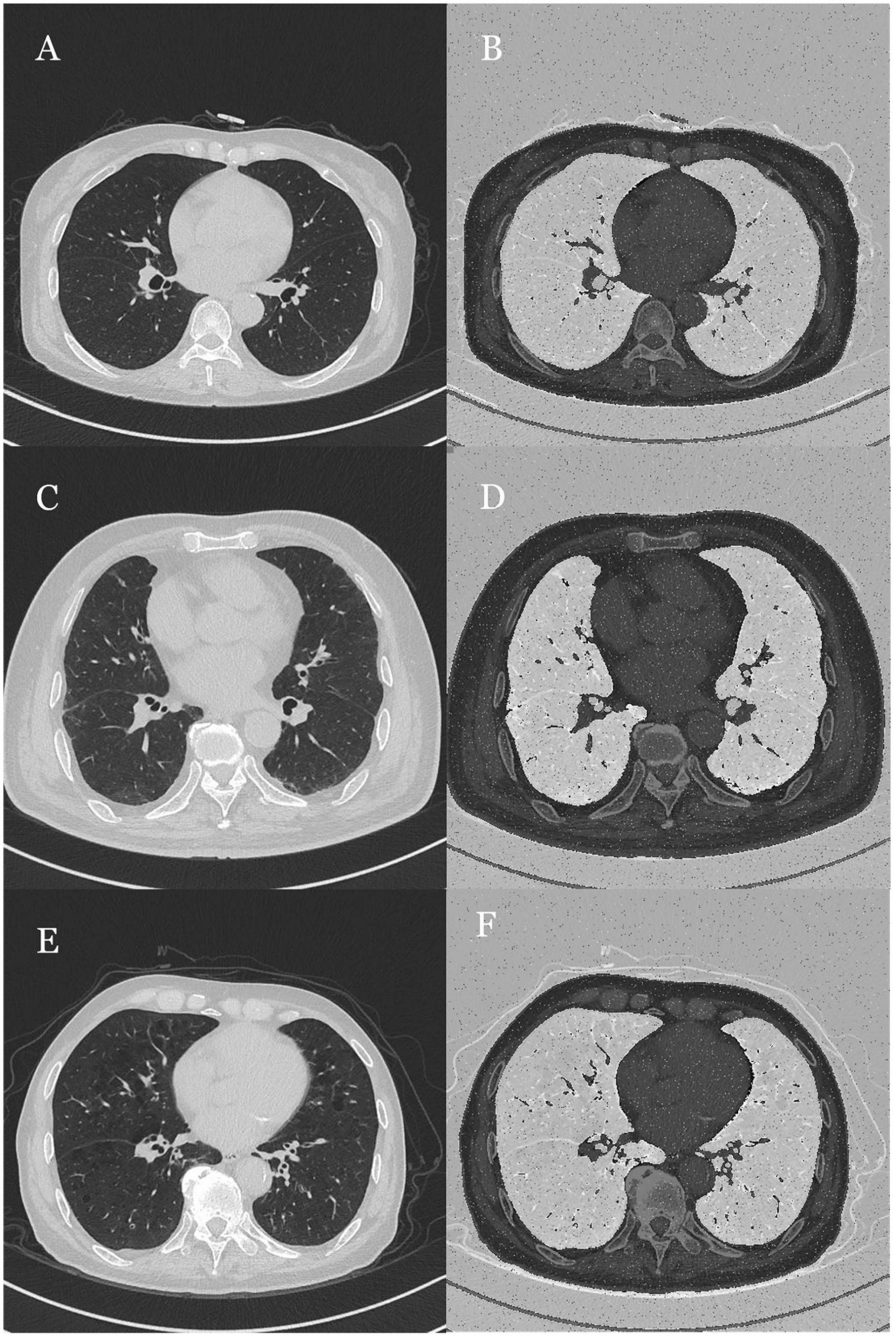
Original and preprocessed chest CT images. **(A)** Original axial chest CT image (lung window) of a normal lung. **(B)** Preprocessed normal chest CT slice after window adjustment (1,500/−450), wavelet denoising (db1), and convolutional denoising autoencoder enhancement. **(C)** Original axial chest CT image (lung window) of a PRISm case. **(D)** Preprocessed PRISm chest CT slice after identical preprocessing. **(E)** Original axial chest CT image (lung window) of a COPD case. **(F)** Preprocessed COPD CT slice after identical preprocessing. All images were standardized to 512 × 512 resolution. PRISm, preserved ratio impaired spirometry; COPD, chronic obstructive pulmonary disease.

To further reduce image noise while preserving important structural details, denoising was performed using the db1 wavelet transform. This approach effectively suppressed random noise artifacts in thin-slice CT images while maintaining tissue boundaries and fine pulmonary textures. All CT slices were then resized to a uniform resolution of 512 × 512 pixels to ensure consistent model input dimensions. Finally, a pre-trained convolutional denoising autoencoder was applied for secondary image enhancement, and the entire dataset was standardized to optimize feature distribution for deep learning model training. Representative examples of the original and preprocessed CT images are shown in Figure 2.

**Figure 3:**
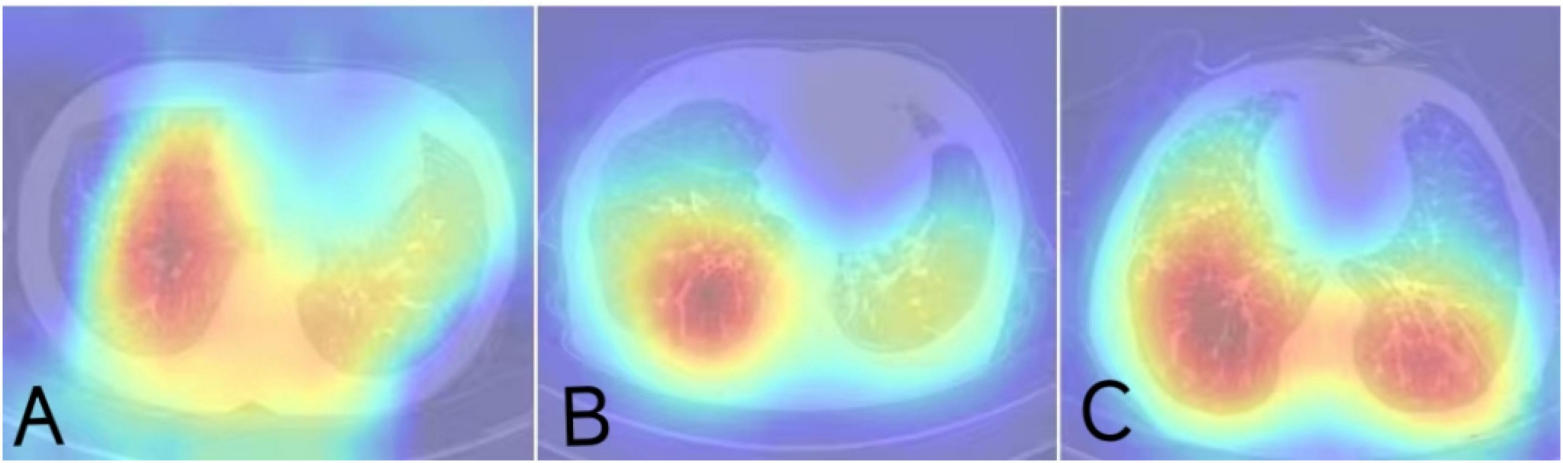
Grad-CAM visualization of representative chest CT slices. **(A)** Normal subject; **(B)** PRISm subject; **(C)** COPD subject. Representative axial CT slices are overlaid with Grad-CAM heatmaps to illustrate the regions contributing most strongly to model predictions. Warmer colors indicate higher attention weights. The visualizations demonstrate that the model primarily focuses on pulmonary parenchymal regions relevant to disease classification rather than unrelated extrapulmonary structures or imaging artifacts. PRISm, preserved ratio impaired spirometry; COPD, chronic obstructive pulmonary disease.

To improve the interpretability of the proposed framework, Grad-CAM visualization analysis was additionally performed on representative CT slices, as illustrated in Figure 3. The visualization results demonstrated that the model primarily focused on lung parenchymal regions relevant to disease classification rather than unrelated background structures such as the chest wall, mediastinum, scanning bed, or image boundary artifacts. These findings indicate that the deep learning framework learned meaningful pulmonary imaging features instead of relying on non-anatomical contextual information. Qualitative inspection of the Grad-CAM heatmaps further revealed increased attention to low-attenuation pulmonary parenchymal regions and airway-related structures, which are commonly associated with airflow limitation, emphysematous changes, and early COPD-related abnormalities. In PRISm cases, the highlighted regions were generally more diffuse and heterogeneous, consistent with the intermediate and transitional nature of this phenotype.

Furthermore, the Grad-CAM heatmaps revealed that PRISm-related imaging characteristics often appeared as diffuse or regional patterns rather than as single clearly delineated focal lesions. Therefore, the generated heatmaps should be interpreted as coarse class-level attribution maps rather than precise lesion segmentation results. Nevertheless, the visualization findings support the biological plausibility and clinical interpretability of the proposed framework by demonstrating that the model predominantly relies on pulmonary parenchymal information for classification.

### 2.5 Demographic data preprocessing

The dataset used in this study included chest CT images together with demographic features. The demographic data comprised sex, age, height, weight, and BMI.

During the data preprocessing phase, each feature was validated for accuracy and completeness. Specific normalization strategies were applied to different variables: sex was encoded as binary values (male = 1, female = 0); age was normalized by dividing by 100; height and weight were standardized by dividing by 200 and 100, respectively; and BMI was normalized by dividing by 40. These normalization procedures ensured that all feature values were distributed within comparable numerical ranges, thereby preventing variables with larger magnitudes from disproportionately influencing the model during training. Finally, all demographic features were integrated into a structured feature vector and combined with CT imaging features to serve as non-imaging inputs for the subsequent multimodal deep learning framework.

### 2.6 Comparison of CT-only deep learning architectures

To comprehensively evaluate the performance of the proposed CT-only model (attention-driven MIL-R34), three mainstream deep learning architectures were selected as comparative baselines: a standard CNN baseline model employing a three-layer convolutional structure followed by fully connected layers 256-3, EfficientNet utilizing a mobile inverted bottleneck convolutional design (fully connected layers 1,280-3), and Swin Transformer based on shifted window attention mechanisms. All four CT-only models maintained an input resolution of 512 × 512, adopted a 6:2:2 data split strategy, and were trained using the AdamW optimizer with ReduceLROnPlateau adaptive learning rate scheduling and early stopping mechanisms.

### 2.7 Model construction of deep learning architectures

**Figure 4:**
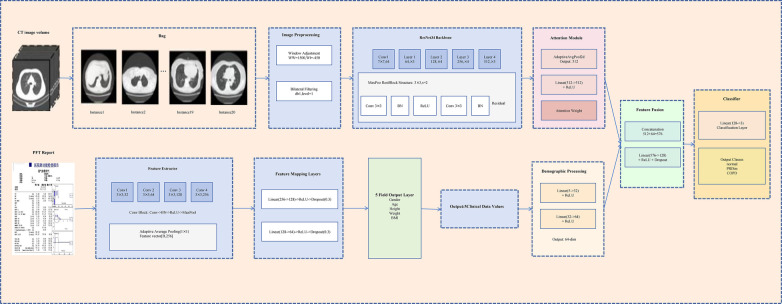
Overall model architecture diagram.

Multimodal converged network design: the model is composed of two parallel branches: (1) an imaging feature extraction branch that uses an enhanced ResNet34 backbone to derive deep features from CT images, with an attention mechanism to emphasize critical feature areas; and (2) a clinical feature processing branch that extracts features from demographic data (age, gender, height, weight and BMI). The features from both branches are concatenated and fed into a joint classifier for final disease classification. The multimodal employs a dual-branch network structure that achieves accurate classification through synergistic analysis of CT imaging features and patient clinical data (Figure 4).

### 2.7 Loss function design

This study employed a composite loss function integrating classification loss and feature separation loss to simultaneously optimize pulmonary disease classification accuracy and feature discriminability. The combined function comprises two components: Focal Loss addresses class imbalance among normal, PRISm, and COPD categories by down-weighting easy-to-classify samples and focusing on challenging cases (12, 13) Loss-F enhances feature space separability through clustering mechanisms that minimize intra-class distances while maximizing inter-class margins for CT imaging and clinical features (14). Guided by multi-task learning principles (15, 16), the final combined loss adaptively weights both components, enabling the multimodal fusion framework to improve diagnostic performance while optimizing discriminative feature representation.

### 2.8 Statistical analysis

All analyses were performed using R software (version 4.2.1; R Foundation for Statistical Computing, Vienna, Austria) and Python's scikit-learn library (version 1.1.2; scikit-learn). Continuous variables were presented as median (interquartile range, IQR) for non-normally distributed data, while categorical variables were presented as *n* or *n* (%). Categorical variables were compared using the chi-square test, while continuous variables were analyzed using the Kruskal–Wallis test (a non-parametric test for comparing three or more groups), followed by Bonferroni *post-hoc* tests for pairwise comparisons. All statistical tests were two-tailed with a significance level set at *P* < 0.05. The diagnostic performance was evaluated through receiver operating characteristic (ROC) curve analysis, calculating the area under the curve (AUC) with 95% confidence intervals.

## 3 Results

### 3.1 Demographic data

A total of 776 participants were included in this study, comprising 196 normal controls, 366 patients with PRISm, and 214 patients with COPD. These participants were randomly allocated into a training set (*n* = 465), a validation set (*n* = 154), and a test set (*n* = 157) at an approximate ratio of 6:2:2. A total of 190 participants were included in the external validation cohort, comprising 45 normal controls, 73 patients with PRISm, and 72 patients with COPD. The demographic characteristics are summarized in Table 1.

**Table 1:** Comparison of baseline data between normal, PRISm and COPD participants.

Characteristic	Training cohort *n* = 465			Validation cohort *n* = 154			Test cohort *n* = 157			*P*-value
	Normal *n* = 118	PRISm *n* = 219	COPD *n* = 128	Normal *n* = 39	PRISm *n* = 73	COPD *n* = 42	Normal *n* = 39	PRISm *n* = 74	COPD *n* = 44	
Age (years)	60.0 (46.2,68.0)	69.0 (62.5,76.0)	71.0 (67.0,76.0)	60.0 (52.5,65.5)	70.0 (64.0,77.0)	70.0 (67.0,74.0)	60.0 (44.5,68.0)	69.5 (62.2,78.0)	71.5 (67.8,76.2)	<0.001
Sex [*n* (%)]										<0.001
Male	64 (54.2%)	134 (61.2%)	110 (85.9%)	18 (46.2%)	45 (61.6%)	37 (88.1%)	20 (51.3%)	40 (54.1%)	36 (81.8%)	
Female	54 (45.8%)	85 (38.8%)	18 (14.1%)	21 (53.8%)	28 (38.4%)	5 (11.9%)	19 (48.7%)	34 (45.9%)	8 (18.2%)	
Height (cm)	166.0 (160.0,172.0)	165.0 (160.0,170.0)	170.0 (165.0,173.0)	166.0 (160.0,170.0)	165.0 (160.0,172.0)	170.0 (163.2,173.8)	167.0 (158.5,172.5)	163.5 (158.2,168.0)	170.0 (169.8,175.0)	<0.001
Weight (kg)	65.0 (55.0,73.0)	63.0 (57.0,72.0)	65.0 (57.8,70.0)	65.0 (60.0,72.0)	65.0 (57.0,72.0)	62.0 (55.5,70.0)	65.0 (58.5,75.0)	60.0 (55.0,69.0)	65.0 (60.0,67.2)	0.682
BMI (kg/m^2^)	23.4 (21.1,25.4)	23.4 (20.8,26.0)	22.5 (20.8,24.5)	23.6 (22.0,25.5)	23.4 (21.2,25.7)	21.9 (19.0,24.7)	23.9 (22.1,26.8)	23.2 (20.5,25.5)	22.1 (20.0,23.9)	0.077
FEV₁ % predicted	105.2 (99.2,115.2)	70.3 (63.2,74.6)	47.4 (34.6,60.2)	105.0 (93.7,112.5)	70.3 (61.2,74.8)	45.5 (34.3,58.6)	104.1 (99.6,112.7)	69.2 (59.1,73.5)	46.0 (37.8,52.1)	<0.001
FEV₁/FVC (%)	89.6 (84.5,94.3)	86.1 (79.6,92.4)	58.1 (46.8,63.3)	88.2 (83.6,91.2)	86.1 (78.6,91.5)	52.0 (46.6,60.4)	90.0 (84.7,94.4)	86.5 (79.9,92.8)	51.7 (44.9,58.1)	<0.001
VC % predicted	97.6 (88.6,105.2)	66.1 (60.1,71.7)	69.3 (59.2,82.1)	95.8 (86.5,100.0)	66.1 (61.8,74.7)	77.4 (56.0,87.4)	96.7 (88.7,102.8)	66.1 (56.3,72.7)	69.3 (61.1,82.3)	<0.001
FEF₅₀ % predicted	105.5 (87.6,128.9)	53.5 (45.0,65.4)	18.4 (12.3,25.9)	102.3 (84.3,109.3)	53.5 (43.6,59.4)	16.4 (11.5,23.9)	105.5 (85.4,126.4)	53.5 (39.4,62.6)	17.4 (11.5,18.9)	<0.001
FEF_75_ % predicted	112.2 (89.3,145.2)	61.4 (48.0,73.7)	23.4 (18.3,32.7)	104.0 (72.5,137.1)	61.4 (50.4,83.2)	22.4 (16.9,30.9)	112.2 (85.0,152.3)	61.4 (48.2,81.8)	23.4 (17.1,25.1)	<0.001
FEF₂₅₋₇₅ % predicted	101.8 (86.6,127.2)	46.2 (46.2,46.2)	19.2 (13.8,27.2)	101.2 (79.2,109.9)	46.2 (46.2,46.2)	16.2 (12.3,27.1)	102.7 (85.3,125.1)	46.2 (46.2,46.2)	17.2 (13.8,19.2)	<0.001
TLC % predicted	88.3 (82.8,94.1)	77.7 (77.7,77.7)	77.2 (73.3,79.0)	88.3 (78.1,91.2)	77.7 (75.8,77.7)	77.2 (75.6,83.3)	88.3 (83.3,92.0)	77.7 (77.7,78.7)	77.2 (71.7,77.2)	<0.001
RV % predicted	113.1 (101.1,122.5)	103.9 (103.9,103.9)	112.2 (104.2,123.6)	104.1 (98.4,115.9)	103.9 (103.9,103.9)	112.2 (103.1,119.1)	113.1 (102.0,128.2)	103.9 (103.9,103.9)	112.2 (107.7,113.7)	<0.001
RV/TLC (%)	124.2 (112.7,136.9)	134.0 (134.0,134.0)	151.3 (143.2,155.7)	124.2 (114.8,131.7)	134.0 (134.0,134.0)	151.2 (136.0,151.3)	125.2 (120.7,137.7)	134.0 (134.0,134.0)	151.3 (137.7,151.3)	<0.001
DLCO % predicted	86.4 (78.0,94.5)	63.8 (63.8,63.8)	35.2 (31.8,40.4)	86.4 (81.0,93.8)	63.8 (62.1,63.8)	35.2 (35.1,44.7)	89.4 (81.2,99.0)	63.8 (63.8,63.8)	35.2 (35.2,35.9)	<0.001
DLCO/VA (%)	101.7 (90.7,113.9)	90.6 (90.6,90.6)	50.3 (45.5,55.1)	103.4 (98.5,113.6)	90.6 (90.6,90.8)	50.3 (50.3,66.7)	108.5 (94.0,116.5)	90.6 (87.6,90.6)	50.3 (50.3,56.2)	<0.001

Data are presented as n (%) for categorical variables and median (first quartile, third quartile) for continuous variables.

Abbreviations: PRISm, preserved ratio impaired spirometry; COPD, chronic obstructive pulmonary disease; FEV_1_, forced expiratory volume in one second; FVC, forced vital capacity; VC, Vital capacity; FEF_50_, Forced Expiratory Flow at 50% of FVC; FEF_75_, Forced Expiratory Flow at 75% of FVC; FEF_25–75_, Forced Expiratory Flow between 25% and 75% of FVC; TLC, Total Lung Capacity; RV, Residual Volume; DLCO, Diffusing Capacity of the Lung for Carbon Monoxide; VA, Alveolar Volume.

Significant differences were observed among the three groups in terms of age, sex distribution, and BMI (all *P* < 0.05), with older age and lower BMI trends observed in the COPD group.

For pulmonary function, significant differences were observed among the three groups (all *P* < 0.05). Normal controls demonstrated the best respiratory status, while the COPD group exhibited the most severe impairment, and the PRISm group showed intermediate changes.

Pulmonary function parameters reflecting small airway function, including FEF₅₀ % predicted, FEF_75_ % predicted, and FEF₂₅₋₇₅ % predicted, also differed among groups, with more pronounced impairment in the COPD group. In addition, the COPD group showed higher RV % predicted and RV/TLC, indicating air trapping and hyperinflation, while diffusion capacity (DLCO % predicted and DLCO/VA) was reduced in both PRISm and COPD groups.

The demographic and clinical characteristics of the external validation cohort are summarized in Table 2. The external cohort exhibited a distribution of demographic and pulmonary function characteristics comparable to that of the internal cohort, although some variability was observed, reflecting the heterogeneity commonly encountered in cross-institutional datasets.

**Table 2:** Comparison of baseline data between normal, PRISm and COPD participants in the external validation set.

Characteristic	Normal (*n* = 45)	PRISm (*n* = 73)	COPD (*n* = 72)	*P*-value
Age (years)	57.0 (55.0, 64.0)	66.0 (52.0, 70.0)	73.0 (70.0, 77.0)	<0.001
Sex [*n* (%)]	Male: 28 (62.2%) Female: 17 (37.8%)	Male: 30 (41.1%) Female: 43 (58.9%)	Male: 57 (79.2%) Female: 15 (20.8%)	<0.001
Height (cm)	170.0 (162.0, 172.5)	165.0 (160.0, 168.0)	170.0 (170.0, 172.0)	<0.001
Weight (kg)	72.0 (58.0, 75.0)	64.0 (60.0, 73.0)	65.0 (64.5, 65.0)	0.003
BMI (kg/m^2^)	24.3 (22.8, 25.2)	24.0 (23.1, 27.8)	22.4 (22.0, 22.5)	<0.001
FEV₁ % predicted	103.8 (103.1, 103.9)	69.9 (69.9, 69.9)	47.5 (45.8, 49.5)	<0.001
FEV₁/FVC (%)	90.0 (90.0, 90.8)	86.4 (86.4, 86.4)	55.1 (55.0, 55.7)	<0.001
VC % predicted	97.4 (97.4, 99.4)	66.1 (66.1, 66.1)	73.4 (68.8, 74.2)	<0.001
FEF₅₀ % predicted	103.9 (103.9, 103.9)	54.2 (54.2, 54.2)	18.4 (18.0, 18.7)	<0.001
FEF_75_ % predicted	120.2 (118.5, 120.2)	61.9 (61.9, 61.9)	23.7 (22.9, 26.8)	<0.001
FEF₂₅₋₇₅ % predicted	103.8 (103.8, 104.5)	41.5 (41.5, 41.5)	18.5 (18.2, 19.8)	<0.001
TLC % predicted	89.3 (89.3, 93.4)	78.0 (78.0, 78.0)	75.8 (73.6, 75.8)	<0.001
RV % predicted	112.8 (112.2, 115.0)	105.3 (105.3, 105.3)	111.8 (103.6, 115.9)	<0.001
RV/TLC (%)	124.2 (122.8, 124.2)	123.0 (123.0, 123.0)	141.2 (140.8, 141.2)	<0.001
DLCO % predicted	87.8 (87.8, 90.2)	61.8 (61.8, 61.8)	37.7 (36.0, 41.6)	<0.001
DLCO/VA (%)	105.8 (105.8, 105.8)	90.3 (90.3, 90.3)	52.1 (51.2, 55.9)	<0.001

Data are presented as n (%) for categorical variables and median (first quartile, third quartile) for continuous variables.

Abbreviations: PRISm, preserved ratio impaired spirometry; COPD, chronic obstructive pulmonary disease; FEV_1_, forced expiratory volume in one second; FVC, forced vital capacity; VC, Vital capacity; FEF_50_, Forced Expiratory Flow at 50% of FVC; FEF_75_, Forced Expiratory Flow at 75% of FVC; FEF_25–75_, Forced Expiratory Flow between 25% and 75% of FVC; TLC, Total Lung Capacity; RV, Residual Volume; DLCO, Diffusing Capacity of the Lung for Carbon Monoxide; VA, Alveolar Volume.

### 3.2 Comparison experiment of model performance

**Figure 5:**
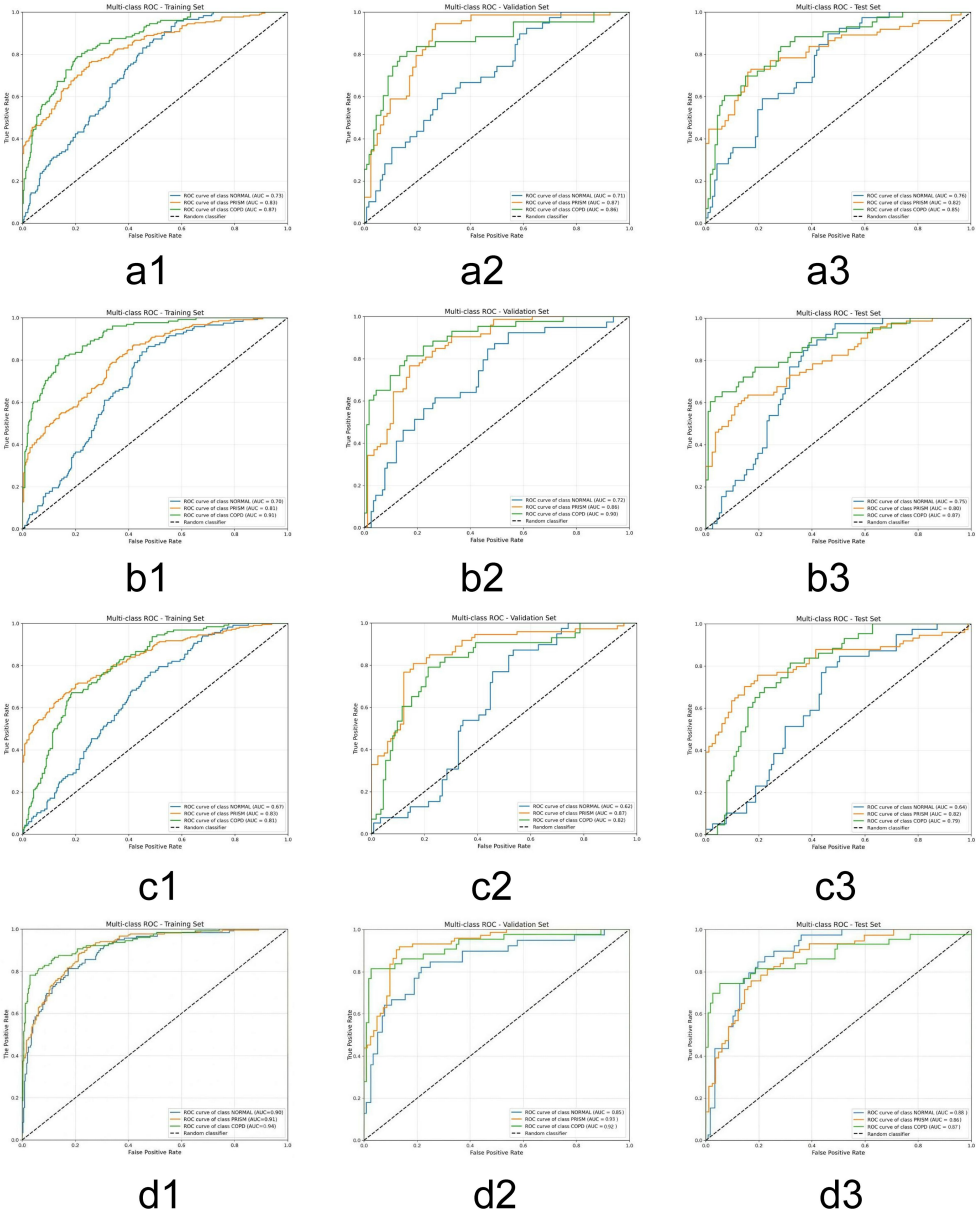
ROC curves for comparative experiments. **(a1-a3)**: M1: CNN– ROC curves for training **(a1)**, validation **(a2)**, and test **(a3)** sets. **(b1-b3)**: M2: SwinTransformer– ROC curves for training **(b1)**, validation **(b2)**, and test **(b3)** sets. **(c1-c3)**: M3: EfficientNet– ROC curves for training **(c1)**, validation **(c2)**, and test **(c3)** sets. **(d1-d3)**: M4: Attention-driven MIL-R34 network – ROC curves for training **(d1)**, validation **(d2)**, and test **(d3)** sets. Each subplot displays three class-specific ROC curves: normal (blue solid line), PRISm (red solid line), and COPD (green solid line), with their corresponding AUC values annotated in the legend. The diagonal black dashed line represents the random classifier baseline (AUC = 0.5). AUC, area under the curve.

The proposed attention-driven MIL-R34 network was compared with three benchmark architectures, including CNN, Swin Transformer, and EfficientNet. As shown in Table 3 and Figure 5, the proposed model achieved the best overall performance among all comparative models on the test cohort. The attention-driven MIL-R34 network attained an accuracy of 0.699 (95% CI: 0.627–0.771), outperforming CNN at 0.641 (95% CI: 0.566–0.716), Swin Transformer at 0.667 (95% CI: 0.593–0.741), and EfficientNet at 0.481 (95% CI: 0.402–0.559).

**Table 3:** Comparative experiments of model performance.

Model	AUC (95% CI)	Accuracy (95% CI)	Sensitivity	Specificity	PPV	NPV
M1: CNN						
Training cohort	0.843 (0.810–0.876)	0.655 (0.612–0.698)	0.656	0.831	0.642	0.825
Validation cohort	0.857 (0.802–0.912)	0.677 (0.604–0.751)	0.668	0.842	0.659	0.835
Test cohort	0.833 (0.775–0.892)	0.641 (0.566–0.716)	0.643	0.824	0.628	0.818
M2: SwinTransformer						
Training cohort	0.808 (0.772–0.844)	0.599 (0.555–0.644)	0.593	0.800	0.597	0.793
Validation cohort	0.830 (0.770–0.889)	0.671 (0.597–0.745)	0.672	0.837	0.670	0.830
Test cohort	0.811 (0.749–0.872)	0.667 (0.593–0.741)	0.679	0.839	0.676	0.831
M3: EfficientNet						
Training cohort	0.769 (0.731–0.807)	0.591 (0.546–0.635)	0.536	0.778	0.629	0.773
Validation cohort	0.769 (0.703–0.836)	0.490 (0.412–0.569)	0.530	0.769	0.629	0.775
Test cohort	0.749 (0.681–0.817)	0.481 (0.402–0.559)	0.513	0.768	0.615	0.769
M4: proposed ResNet34						
Training cohort	0.916 (0.891–0.941)	0.750 (0.711–0.789)	0.768	0.879	0.756	0.872
Validation cohort	0.901 (0.855–0.948)	0.729 (0.659–0.799)	0.759	0.871	0.746	0.865
Test cohort	0.873 (0.821–0.925)	0.699 (0.627–0.771)	0.732	0.854	0.716	0.851

Data are presented as AUC, with 95% confidence intervals in parentheses. AUC, area under the curve; NPV, negative predictive value; PPV, positive predictive value.

In terms of discriminative ability, the proposed ResNet34 model achieved the highest AUC of 0.873 (95% CI: 0.821–0.925), compared with 0.833 (95% CI: 0.775–0.892) for CNN, 0.811 (95% CI: 0.749–0.872) for Swin Transformer, and 0.749 (95% CI: 0.681–0.817) for EfficientNet. Furthermore, the proposed model consistently demonstrated superior performance across additional evaluation metrics, including sensitivity (0.732), specificity (0.854), PPV (0.716), and NPV (0.851), indicating a more balanced and robust classification capability for distinguishing normal, PRISm, and COPD subjects.

Overall, these findings demonstrate that the proposed ResNet34-based framework provides better feature representation and classification performance than the other benchmark architectures in the three-class classification task.

### 3.3 Ablation study conducted in the proposed multimodal

**Figure 6:**
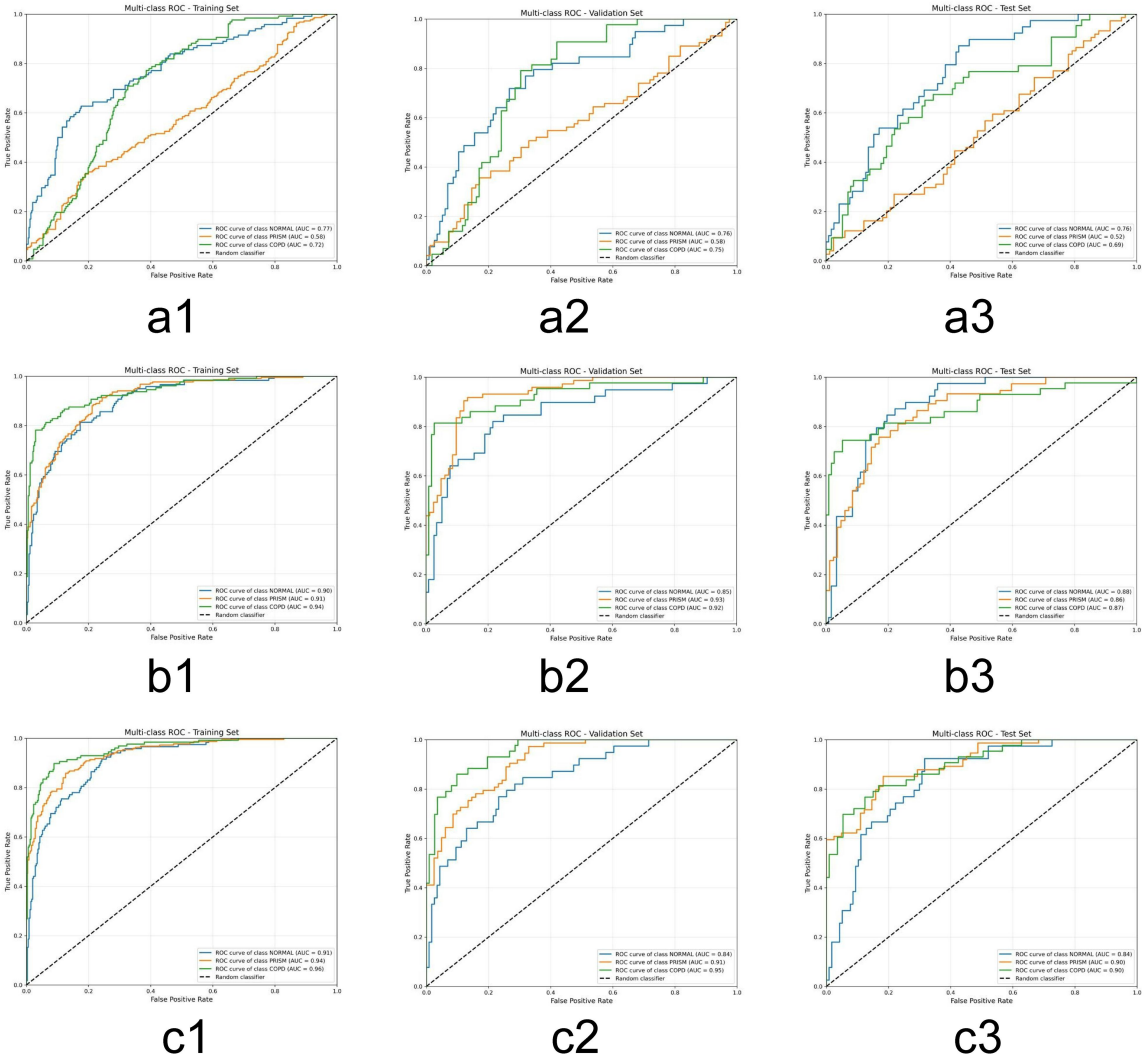
ROC curves for ablation experiments. **(a1-a3)**: M1: demographic – ROC curves for training **(a1)**, validation **(a2)**, and test **(a3)** sets. **(b1-b3)**: M2: CT imaging – ROC curves for training **(b1)**, validation **(b2)**, and test **(b3)** sets. **(c1-c3)**: M3: demographic+CT imaging – ROC curves for training **(c1)**, validation **(c2)**, and test **(c3)** sets. Each subplot displays three class-specific ROC curves: normal (blue solid line), PRISm (red solid line), and COPD (green solid line), with their corresponding AUC values annotated in the legend. The diagonal black dashed line represents the random classifier baseline (AUC = 0.5). PRISm, preserved ratio impaired spirometry; COPD, chronic obstructive pulmonary disease; AUC, area under the curve; NPV, negative predictive value; PPV, positive predictive value.

**Figure 7:**
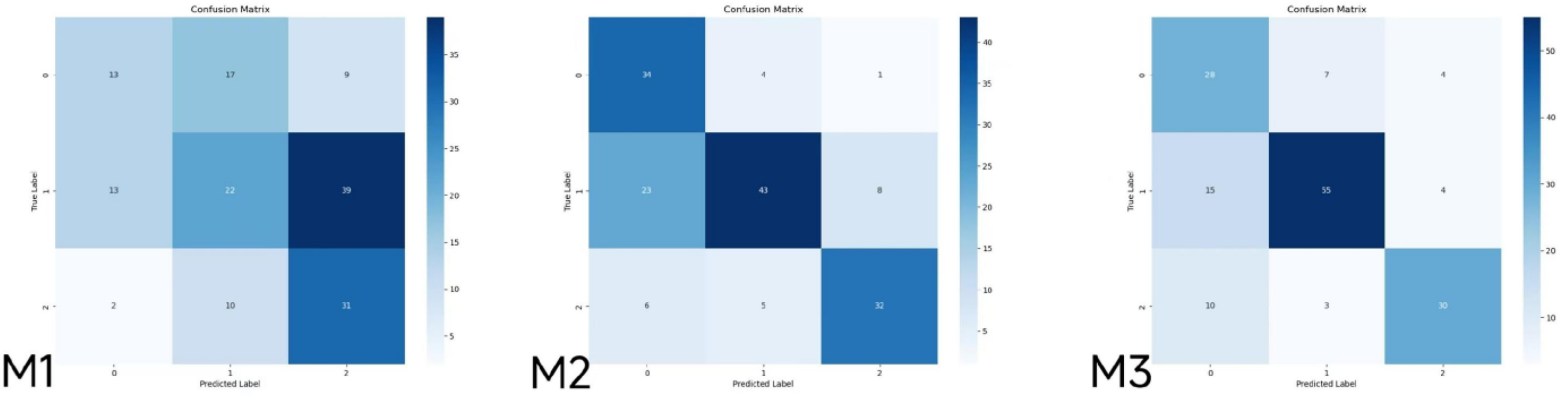
Confusion matrices for ablation experiments on the test set. M1 (demographic only), M2 (CT imaging only), and M3 (demographic + CT imaging). Each subplot displays the classification results for three classes: normal, PRISm, and COPD, with true labels on the *y*-axis and predicted labels on the *x*-axis. The diagonal cells represent correctly classified samples, while off-diagonal cells indicate misclassifications. PRISm, preserved ratio impaired spirometry; COPD, chronic obstructive pulmonary disease.

Ablation experiments were conducted in this study to evaluate the individual contributions of demographic information and CT imaging to the overall classification performance. The ROC curves and confusion matrices for the test cohort are presented in Figure 6 and Figure 7, respectively, and the corresponding quantitative results are summarized in Table 4.

**Table 4:** Ablation study of model performance.

Model	AUC	Accuracy	Sensitivity	Specificity	PPV	NPV
M1: demographic						
Training cohort	0.689 (0.647–0.731)	0.511 (0.465–0.556)	0.550	0.756	0.538	0.764
Validation cohort	0.698 (0.626–0.770)	0.510 (0.431–0.588)	0.548	0.755	0.524	0.767
Test cohort	0.656 (0.582–0.731)	0.423 (0.346–0.501)	0.451	0.706	0.435	0.718
M2: CT imaging						
Training cohort	0.916 (0.891–0.941)	0.750 (0.711–0.789)	0.768	0.879	0.756	0.872
Validation cohort	0.901 (0.855–0.948)	0.729 (0.659–0.799)	0.759	0.871	0.746	0.865
Test cohort	0.873 (0.821–0.925)	0.699 (0.627–0.771)	0.732	0.854	0.716	0.851
M3: demographic+CT imaging						
Training cohort	0.935 (0.912–0.957)	0.795 (0.759–0.832)	0.788	0.897	0.791	0.894
Validation cohort	0.902 (0.855–0.949)	0.742 (0.673–0.811)	0.734	0.869	0.737	0.865
Test cohort	0.879 (0.828–0.930)	0.724 (0.654–0.794)	0.720	0.865	0.721	0.858

Data are presented as AUC, with 95% confidence intervals in parentheses. AUC, area under the curve; NPV, negative predictive value; PPV, positive predictive value.

Using test set accuracy as the primary evaluation metric, the demographic-only model (M1) achieved a test accuracy of 0.423 (95% CI: 0.346–0.501) with an AUC of 0.656 (95% CI: 0.582–0.731), indicating limited discriminative ability when relying solely on demographic features. The CT-only model (M2) substantially improved classification performance, achieving a test accuracy of 0.699 (95% CI: 0.627–0.771) and an AUC of 0.873 (95% CI: 0.821–0.925). This result highlights the strong predictive value of CT imaging features for distinguishing normal, PRISm, and COPD subjects. The multimodal model (M3) which integrates both CT imaging and demographic information, achieved the best overall performance, with a test accuracy of 0.724 (95% CI: 0.654–0.794) and an AUC of 0.879 (95% CI: 0.828–0.930). Overall, these findings demonstrate that CT imaging serves as the primary contributor to classification performance, while the incorporation of demographic information provides complementary value, enhancing model robustness, reliability, and generalization capability in the three-class classification task.

### 3.4 External validation

**Figure 8:**
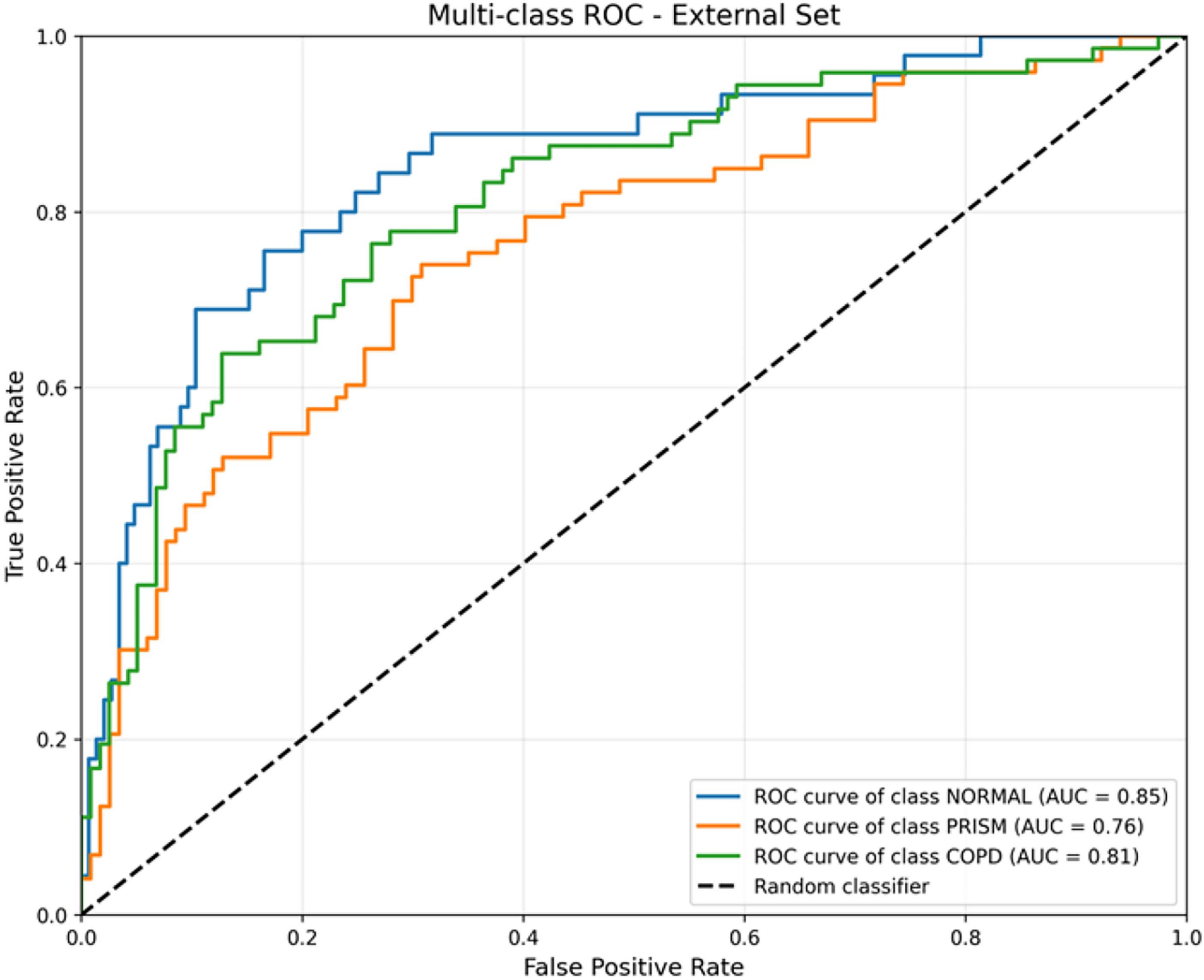
ROC curves for external.

External validation experiments were conducted in this study to assess the generalization capability of the proposed model on an independent cohort. The ROC curves for the external validation cohort are presented in Figure 8, and the corresponding quantitative results are summarized in Table 5.

**Table 5:** Performance metrics of the proposed model on the external validation cohort.

Model	AUC (95% CI)	Accuracy (95% CI)	Sensitivity	Specificity	PPV	NPV
External	0.808 (0.752–0.864)	0.642 (0.574–0.710)	0.631	0.798	0.637	0.793

Using accuracy as the primary evaluation metric, the proposed model achieved an accuracy of 0.642 (95% CI: 0.574–0.710) with an AUC of 0.808 (95% CI: 0.752–0.864) on the external validation dataset. The sensitivity and specificity were 0.631 and 0.798, respectively, while the PPV and NPV were 0.637 and 0.793.

Compared with the internal test cohort, a decrease in performance was observed in the external validation cohort. This reduction is likely attributable to differences in data distribution, imaging protocols, scanner characteristics, and population demographics across institutions. Nevertheless, the model maintained an AUC above 0.80 and an accuracy exceeding 0.64, indicating reasonable discriminative ability and generalization performance under cross-institutional settings.

Notably, most misclassifications occurred in PRISm cases, which is expected given the heterogeneous and transitional nature of PRISm between normal lung function and established COPD. Overall, these findings demonstrate that the proposed multimodal framework retains acceptable robustness and generalizability when applied to independent external data.

## 4 Discussion

The accurate identification of preserved ratio impaired spirometry (PRISm) represents a critical and unmet need in pulmonary medicine. In this study, we developed and validated a multimodal deep learning framework. This is an attention-driven multi-instance learning framework with an ResNet34 backbone that integrates thin-slice chest CT images with demographic information to discriminate among normal subjects, individuals with PRISm, and patients with COPD.

In comparative experiments against three benchmark architectures (standard CNN, Swin Transformer, and EfficientNet), the proposed ResNet34 achieved the best overall performance, with a three-class test accuracy of 0.724 (95% CI: 0.654–0.794) and an AUC of 0.879 (95% CI: 0.828–0.930) (Table 3; Figure 5). This performance consistently exceeded that of CNN (accuracy: 0.641, AUC: 0.833), Swin Transformer (accuracy: 0.667, AUC: 0.811), and EfficientNet (accuracy: 0.571, AUC: 0.793). These findings demonstrate that the ResNet34 backbone provides superior feature representation for distinguishing among the three pulmonary function categories, which facilitate the capture of subtle and heterogeneous imaging abnormalities associated with early-stage lung function impairment.

The ablation study further elucidated the complementary value of CT imaging and demographic information (Table 4; Figures 6, 7). The demographic-only model (M1) achieved limited discriminative ability, with a test accuracy of 0.423 and an AUC of 0.656, indicating that demographic variables alone are insufficient for reliable three-class classification. In contrast, the CT-only model (M2) substantially improved performance, achieving a test accuracy of 0.699 and an AUC of 0.873, confirming that CT imaging features serve as the primary contributor to classification performance. Notably, the multimodal model (M3), which integrates both CT imaging and demographic data, achieved the best overall performance, with a test accuracy of 0.724 and an AUC of 0.879. Compared with the CT-only model, M3 also demonstrated improvements in specificity (0.865 vs. 0.854), PPV (0.721 vs. 0.716), and NPV (0.858 vs. 0.851). These results suggest that demographic information provides complementary contextual information that enhances model robustness and generalization capability. This multimodal design mirrors real-world clinical reasoning, where imaging findings are interpreted together with patient characteristics to support diagnosis and disease assessment.

A key contribution of this study is the adoption of a three-class classification strategy, which more closely reflects real-world clinical scenarios where PRISm represents an intermediate and heterogeneous disease state between normal lung function and established COPD. The confusion matrices in Figure 7 reveal that most misclassifications occurred between PRISm and its neighboring categories (normal and COPD), rather than between normal and COPD directly. This pattern confirms the transitional nature of PRISm and highlights the inherent challenge of distinguishing it from adjacent disease states. Notably, the multimodal model (M3) reduced the misclassification of PRISm cases compared with the CT-only model (Figure 7B vs. Figure 7C), indicating that demographic information helps refine the differentiation of this intermediate phenotype.

The multi-level denoising strategy, combining wavelet transforms (17) with noise-insertion autoencoders (18), played an important role in preserving fine-grained structural information while suppressing noise artifacts. This finding is consistent with previous observations (19) and recent advances in LDCT denoising (20). This preprocessing approach, together with the attention mechanism and the improved ResNet34 backbone, enabled the framework to capture subtle and heterogeneous imaging abnormalities that are often difficult to identify using conventional radiological assessment alone.

In this retrospective analysis, 776 participants from Huadong Hospital and 190 participants from Jinggu County People's Hospital were used for stratified sampling validation, and the LOSS-F function effectively enhanced feature discrimination among the three classes. Compared with previous studies, our research demonstrates several important innovations. First, we successfully implemented a three-class classification framework, overcoming the binary classification limitation of many existing approaches that only distinguish between two categories. Second, the optimized preprocessing and feature extraction strategy effectively addressed noise interference in thin-slice CT imaging. Third, the proposed multimodal framework integrates demographic information with CT imaging features, thereby improving overall classification robustness and clinical applicability. The attention mechanism further enhances interpretability by highlighting the lung parenchymal regions most relevant to the model's predictions, as demonstrated by the Grad-CAM visualizations in Figure 3. Qualitative inspection of the heatmaps showed that the model primarily focused on low-attenuation lung parenchyma and airway-related regions, which are commonly associated with airflow limitation and COPD-related abnormalities.

Despite these promising findings, several limitations should be acknowledged. First, the multimodal achieved the best overall performance, with a test accuracy of 0.724 and an AUC of 0.879. However, external validation on an independent cohort yielded a lower accuracy of 0.642 and an AUC of 0.808, which may be attributable to differences in imaging protocols, scanner characteristics, and population distributions across institutions. Second, although the overall classification performance was encouraging, misclassification between PRISm and adjacent disease states was still observed, reflecting the intrinsic heterogeneity and transitional characteristics of PRISm. Furthermore, smoking history and respiratory symptom variables were not consistently available across all cohorts and therefore could not be incorporated into the current framework. Future studies may expand the sample size and incorporate additional clinical variables, radiomics signatures, or serum biomarkers to enhance the model's ability to classify normal, PRISm, and COPD (21). In addition, future research should prioritize large-scale prospective validation to further establish the robustness and real-world applicability of the proposed framework.

## 5 Conclusions

We developed a multimodal attention-driven MIL framework with an improved ResNet34 backbone (MIL-R34) for three-class classification of normal, PRISm, and COPD using chest CT imaging and clinical data. The multimodal approach outperformed unimodal models and captured the transitional nature of PRISm. This proposed framework offers a promising tool for early risk stratification and targeted intervention.

## Data Availability

The raw data supporting the conclusions of this article will be made available by the authors, without undue reservation.
